# The Effect of Cytotoxicity and Antimicrobial of Synthesized CuO NPs from Propolis on HEK-293 Cells and *Lactobacillus acidophilus*

**DOI:** 10.1155/2023/1430839

**Published:** 2023-02-09

**Authors:** Yasamin Seyyed Hajizadeh, Ebrahim Babapour, Naser Harzandi, Mohsen Yazdanian, Reza Ranjbar

**Affiliations:** ^1^Department of Microbiology, Karaj Branch, Islamic Azad University, Karaj, Iran; ^2^Research Center for Prevention of Oral and Dental Diseases, Baqiyatallah University of Medical Sciences, Tehran, Iran; ^3^Molecular Biology Research Center, Systems Biology and Poisonings Institute, Baqiyatallah University of Medical Sciences, Tehran, Iran

## Abstract

**Background:**

Drug resistance is currently possible anywhere in the world. Due to the discovery of antimicrobials, medicine, and health have made tremendous advances over the past several decades.

**Aim:**

This research evaluated the antimicrobial and cytotoxicity effects of green synthesis of copper oxide nanoparticles (CuO NPs) on *Lactobacillus acidophilus* and human embryonic kidney 293 cells (HEK). *Method and Materials*. Propolis was sampled and extracted. Green synthesis of CuO NPs was synthesized and characterized using SEM, TEM, DLS, BET, and zeta potential methods. *L. acidophilus* (ATCC 4356) was used, and the antimicrobial tests were carried out at different concentrations (10≥ mg/ml). Moreover, the cytotoxicity was evaluated using an MTT assay on human embryonic kidney 293 cells (HEK).

**Results:**

Synthesized CuO NPs using propolis extracts from Khalkhal (sample 1) and Gillan (sample 2) showed −13.2 and −14.4 mV, respectively. The hydrodynamic sizes of well-dispersed samples 1 and 2 were 3124.9 nm and 1726.7 nm, respectively. According to BET analysis, samples 1 and 2 had 5.37 and 8.45 m^2^/g surface area, respectively. The surface area was decreased due to the addition of propolis extract, and the pore size was increased. CuO NPs of samples 1 and 2 were visible on SEM images with diameters ranging from 75 to 145 nm and 120 to 155 nm, respectively. Based on TEM analysis, the size of CuO particles was increased in samples 1 and 2. CuO NPs particles had narrow size distributions with evenly dispersed NPs on all sides. The cell viability of the CuO NPs of samples 1 and 2 after 24, 48, and 72 hours was greater than 50%. As a result of the MIC and MBC tests, it was determined that samples 1 and 2 had the same effect against *L. acidophilus* (0.0024 mg/ml). Biofilm formation and degradation of sample 1 were more efficient against *L. acidophilus*.

**Conclusion:**

There was no evidence of cytotoxicity in the samples. In addition, results showed that the green synthesized CuO NPs from Khalkhal propolis were effective against *L. acidophilus*. Thus, the green synthesized CuO NPs from Khalkhal propolis were the best candidates for clinical application.

## 1. Introduction

Microorganisms in the mouth can destroy teeth. Throughout the world, this disease affects many generations. In recent years, silver metal has been replaced with resin restorations due to the advent of silver amalgam treatments in dentistry in the 19th century [1]. During the last few decades, antibiotics have made considerable advances in the medical field. An emerging phenomenon called antimicrobial resistance could impede international health and sustainable development goals by compromising therapeutic strategies. As a result of AMR, countries and continents have been affected more rapidly than ever, making it one of the most severe public health crises ever. Antibiotic Stewardship program activities include ensuring appropriate diagnostics and treatment of drug-resistant infections and appropriate use of antibiotics judiciously [2]. There is no doubt that nanotheranostics represents an advancement in nanomedicine. Nanomaterials and nanotechnology can be used to improve medicinal Efficacy. A unique physicochemical property, targeted delivery, and reduced chance of developing resistance make NPs a popular alternative to antibiotics. Metallic NPs are most commonly reported as alternative antibacterial agents [2–5]. *Lactobacillus* is acidogenic bacteria that cause dental caries. Children and adults suffer from pain, structural damage to their teeth, and eventual tooth loss due to the loss of mineralized tissues. *Lactobacillus* is the most common bacteria that cause periodontal diseases and cause inflammation, and infection [6–11]. An arsenal of redox potentials is found in plant extracts. NPs with defined sizes can be produced by biogenic synthesis using phytochemical compounds as stabilizers. Propolis is used in traditional medicine for its biological properties. The antioxidative, anti-inflammatory, and antibacterial properties of propolis are noteworthy. Propolis exhibits antiproliferative and antitumor properties *in vitro* and *in vivo*. It depends on the region, climate, and extraction season. Because propolis contains polyphenolic acids, flavonoids, and terpenoids, it can produce gold NPs [12–18]. The cost of manufacturing metallic NPs can be reduced by capping and reducing metallic NPs with biological components. Since they do not require excessive pressure or energy, they are environmentally friendly and energy-efficient [18–21]. NPs can be manufactured from medicinal plants. Allium sativum, Aleo vera, and Punica granatum have been used to synthesize copper NPs [22, 23]. This research aimed to synthesize the CuO NPs using propolis extracts and evaluate their antibacterial and cytotoxicity on *Lactobacillus acidophilus* and HEK-293 cells, respectively.

## 2. Method and Material

### 2.1. Materials

**Figure 1:**
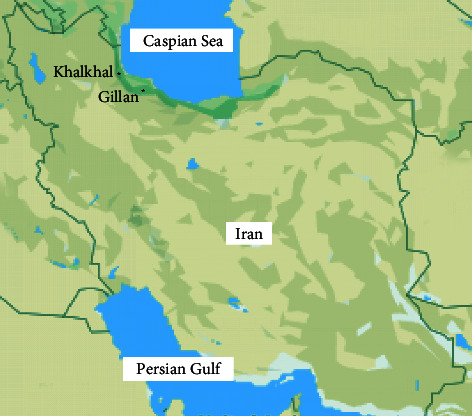
Two propolis samples were collected from Khalkhal, Ardabil province, and Gillan province, Iran.

Propolis was collected from Gillan and Khalkhal (Figure 1). To synthesize CuO NPs, the following materials need to be used: copper sulfate pentahydrate (CuSO_4_.5H_2_O) (Sigma Aldrich, Co., USA) and absolute ethanol (Merck, Germany). This experiment used reagents purchased from Merck, Germany; none of them were purified before use.

### 2.2. Preparation of Propolis Extract

Ground samples were prepared and frozen (−20°C) for a while. In this study, raw propolis samples were extracted (by stirring them in a tetrafold amount of ethanol (70%) for three days in a dark environment at room temperature) to quantify their ethanol content. To remove waxes and other less soluble substances from the suspensions, they were filtered (Whatman paper, No.1) to make sure that all waxes and other less soluble substances were removed from the suspensions. It was necessary to repeat this procedure three times to get the desired results. The freeze-dried extract solution was obtained for the next steps [24].

### 2.3. Green Biosynthesized Copper NPs

To synthesize CuO NPs, the procedure was to dissolve 1 mg of extract in deionized water to form a solution and then adjust the pH of this solution to 8 by adding NaOH to form a solution. A solution of copper sulfide (6 mM) was slowly added to 100 ml of the extract solution while stirring with a rotation speed of 1,000 rpm. The samples were then placed in a flask and stirred (24°h, 37°C to 40°C) in the dark at a temperature between 37°C and 40°C in the dark. After the colored mixture (dark brown) had been obtained, it was centrifuged at 13,000 rpm (15 min, 25°C) to obtain the colored mixture (dark brown). A deionized water wash was performed on the pellet twice to ensure that all residues were removed from the extract. A lyophilized precipitate was then stored to be analyzed in the future.

### 2.4. Zeta Potential Analysis

Zeta Potential analysis was used to detect the hydrodynamic size polydispersity index and the size polydispersity index of the synthesized NPs. Particle sizes were evaluated under an angle of 90 degrees at 25°C.

### 2.5. DLS Analysis

This study used the DLS method to size metal oxide NPs (measured in terms of hydrodynamic radius) according to the range sizing method. An open capillary cell was used in a 37°C water bath, and a disposable folded capillary cell was used to measure the particle sizes of the dispersions after they had been prepared using a disposable folded capillary cell.

### 2.6. BET Analysis

As a part of the investigation, the surface area of NPs under vacuum was determined using the Brunauer–Emmett–Teller (BET). As part of the surface area measurement, a 2-hour vacuum degassing of the sample was carried out at 120°C.

### 2.7. SEM Analysis

To determine the structure and morphology structure of the NPs, the SEM examination was carried out. As part of the SEM studies, approximately 25 *μ*l of NPs were applied to the CuO stub, and the results were evaluated. Using a scanning electron microscope, photographs of the samples were taken [25]. Approximately 10 kV of accelerating voltage was used with a working distance of approximately 3 mm. The brightness and contrast correction also resulted in clear and distinguishable images that could be displayed. The lengths of the measurement scales, 500 nm and 20 *μ*m, were calibrated using the NISTRM for calibration purposes.

### 2.8. TEM Analysis

The shapes of the CuO NPs were observed using TEM imaging based on suspensions induced by propolis-mediated suspensions accelerated at 200 KV and tilted at X-tilt ± 60°. A copper grid coated with carbon was used as a platform for the droplet of NPs solutions to achieve this goal. A specimen holder was used to mount the grid once it had dried, and a few minutes later, it was mounted on the specimen holder for drying. The TEM image of the selected area of the sample was overlaid with the selected area's diffraction pattern to clarify the sample's lattice pattern and crystallite size [26].

### 2.9. Cytotoxicity Assay

Biosynthesized NP solutions were sonicated before using in any experiments. The NPs were sterilized using UV light. In the culture of the human embryonic kidney 293 cells (HEK), 100 U/mL penicillin, 10% fetal bovine serum (Himedia), 100 U/mL penicillin, and 2 mM L-glutamine were used. The cells were maintained at 70–80% confluency by passage every 2–4 days in a 25 cm^2^ flask [27]. Human embryonic kidney 293 cells (HEK) were used to test the green biosynthesized NPs [28–31]. In 96-well plates, 100 *μ*l of cell suspension was filled into each well, containing 10000 cells. A set of parameters was followed during incubation. The concentrations of CuO NPs were 2.5, 5, 10, 25, 50, 75, and 100 *μ*g/mL, and the control group was cell culture. Once the initial model had been dissolved in DMSO, the NPs were dispersed in PBS. After adding the samples, the plates were incubated for 48 hours. A control medium was used without test samples. Each well was incubated for four hours at 37°C with 15 *μ*l of MTT in PBS. We dissolved the formazan crystals in 100 *μ*l of DMSO after removing the MTT medium and the medium containing MTT. 570 nm measurements were taken to determine the absorbance. The cell viability percentage was calculated using the following equation:

(1) $$\begin{array}{c} Cell\;Viability\%=\frac{sample}{control}\times 100\text{.} \end{array}$$

### 2.10. Determination of Minimal Inhibitory/Bactericidal Concentrations (MIC/MBC)

This experiment used *Lactobacillus acidophilus* (ATCC 4356) as the bacteria. 1.5 × 10^8^ CFU/mL was the final concentration [32]. MICs and MBCs of green synthesized CuO NPs from Khalkhal (sample 1) and Gillan (sample 2) propolis were determined following the guidelines of the NCCLS. To prepare the nutrient broth medium for dilution, a concentration of 10 mg/mL of CuO NPs was added to the nutrient broth at 10 mg/mL. To create solid nutrient agar plates, solid nutrient agar was used for plating the bacteria. It was determined that an inhibitory concentration was reached when the lowest concentration inhibited bacterial growth. The MBC needs to be determined in a concentration that allows bacterial growth to be inhibited entirely. Our experiments were repeated in equal numbers for each sample. The average growth rate of the bacteria on each plate was calculated based on the average growth rate on each plate [33].

### 2.11. Biofilm Formation Analysis

In the process of conducting this test, microdilutions were used as a dilution method. It was observed that bacteria were cultured for 24 hours using TSB. TSB containing 1% sucrose was used as the dilution solution, and the suspension was diluted 1 : 100 in the solution. The extracts containing 10× MBC were used for this test. To transfer 100 *μ*l of a mixture of bacterial suspension and extract solution to each of the microplate wells, 200 *μ*l of 100 *μ*l of a suspension of pathogenic bacteria was transferred, 100 *μ*l of extract solution, and 100 *μ*l of mouthwash. As a positive control, 0.2% chlorhexidine was added as a solution to 200 *μ*l of bacterial suspension and physiological saline in which bacterial suspension and physiological saline were mixed. During the incubation period of 24 hours, the plate surface was incubated at 37°C in an incubator. Using a buffer containing phosphate and saline, the wells were washed three times with the solution after the contents had been removed from them. It was necessary to do this to remove disconnected cells. In the following step, 200 *μ*l of 33% glycolic acetic acid was added to the wells containing cells that had adhered to the bottom to remove them. After 15 minutes, the optical density at 570 nm of each well of each sample was measured using an ELISA reader, and biofilm formation rates (%) were calculated using the mentioned formula based on the optical density at 570 nm of each sample.

(2) $$\begin{array}{c} The\;biofilm\;formation\;rate=\frac{Samples\left(OD\right)}{Control\left(OD\right)}\times 100\text{.} \end{array}$$

OD treatments and OD controls are defined as the absorbance at 570 nm in each well after the dissolving solution was added to the well with and without the sample, respectively.

### 2.12. The Biofilm Degradation Analysis

This test was also conducted to investigate whether microdilution has any destructive effects on biofilms. Microplates were inoculated with bacteria using TSB medium, 3% glucose, and synthetic saliva (McDougall solution) for growth. A biofilm was formed as soon as the remaining culture medium of the culture was discarded. The MBC solution was diluted ten times with the sample solution. To remove the biofilms on the walls of the wells, phosphate buffer was used. After applying it to them, it took 15 minutes for the walls to become saturated with 1% violet crystal. Sterile solutions of water mixed with 95% alcohol were used to clean the wells, and three rinses of sterile water were used after each. After the suspension was transferred from one microplate to another, it was incubated for 45 minutes before the new microplate was used. Using a microplate reader at a wavelength of 570 nm, we measured the optical density of the suspension in each well to determine the extent of degradation of the biofilm. The positive control was chlorhexidine 0.2%. Through the use of the equation mentioned, we were able to calculate the percentage rate of biofilm degradation using the following equation [34]:

(3) $$\begin{array}{c} The\;biofilm\;degradation\;rate=100-\left(\frac{Samples\left(OD\right)}{Control\left(OD\right)}\right)\times 100\text{.} \end{array}$$

OD treatments and OD controls are defined as the absorbance at 570 nm in each well after the dissolving solution was added to the well with and without the sample, respectively.

### 2.13. Statistical Analysis

It was performed independently in triplicates for each of the tests. A one-way ANOVA test (SPSS statistics model 20) was used to compare the means among the groups, and Tukey's post hoc test allowed further comparisons. The significance level was *P* value <0.05.

## 3. Results

### 3.1. Characterization Results

A UV-visible spectrophotometer was used in a previous study and performed surface plasmon resonance measurements on CuO NPs. In UV-Vis spectra of CuO NPs prepared from propolis (Khalkhal) extract, a characteristic peak at 385 nm can be seen on the spectrum. As a result of further UV-Vis spectrophotometry investigation into CuO NPs using propolis extract (Gillan), peaks are observed at 243, 292, and 350 nm. CuO NPs were analyzed using XRD techniques using extracts of propolis (Khalkhal), resulting in crystallographic planes of face-centered cubic (FCC) with peaks of diffraction around 2*θ* = 35.74°, 39.04°, and 49.04°. The diffraction peaks of CuO NPs using extracts of propolis (Gillan) were observed around 2*θ* = 25.54°, 26.69°, 38.79°, and 48.84°. The Khalkhal propolis extract FTIR spectrum showed a sharp peak at 3422 cm^−1^ due to free hydroxyl groups and their intramolecular and intermolecular hydrogen bonds. Sharp peaks at 2925, 1637, and 1515 to 1076 cm^−1^ were associated with C=O and C=C aromatic stretching frequencies. CuO NP monoclinic phase exhibits an absorption band of 602 cm^−1^. As a result of free hydroxyl groups and their intramolecular and intermolecular H-bonds, CuO NPs of the Gillan propolis extract spectrum also peak at 3410 cm^−1^. CSp3-H and aromatic stretching frequencies of C=O and C=C were related to the peaks at 2920, 1614, and 1515 to 1057 cm^−1^. An absorption band of 603 cm^−1^ was observed in the monoclinic phase of CuO NPs [35].

### 3.2. Zeta Potential DLS Analysis

**Figure 2:**
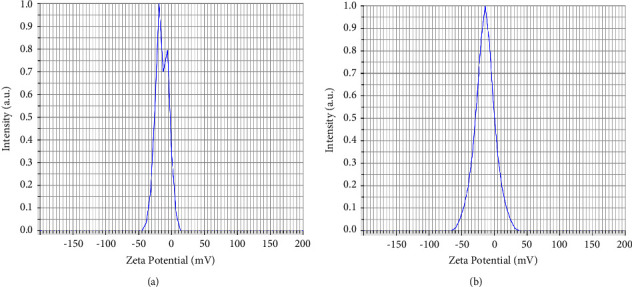
Zeta potential of the synthesized CuO of (a) samples 1 (Khalkhal) and (b) sample 2 (Gillan).

**Figure 3:**
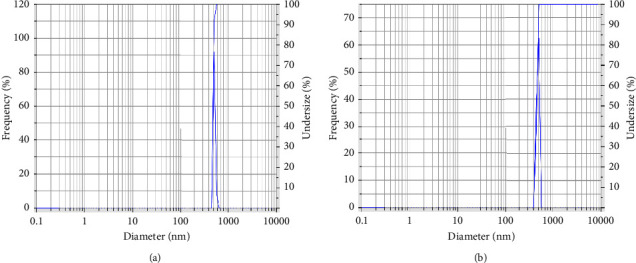
Size distributions of the synthesized CuO of (a) samples 1 (Khalkhal) and (b) sample 2 (Gillan).

CuO NPs produced from Khalkhal propolis extract (sample 1) showed a zeta potential of −13.2 mV. In contrast, the produced CuO NPs from Gillan propolis extract (sample 2) had a zeta potential of −14.4 mV (Figure 2). As a result of using the sonication method provided by the National Institute of Standards and Technology (NIST), the CuO NPs were separated from CuO NPs synthesized from Khalkhal (sample 1) and Gillan propolis extracts (sample 2); however, there was no noticeable difference between the well-synthesized CuO NPs synthesized using propolis extract and those synthesized using propolis extracts. For well-dispersed samples 1 and 2, it was found that the hydrodynamic sizes were 3124.9 nm and 1726.7 nm, respectively, based on DLS data (Figure 3).

### 3.3. BET Evaluation

Table 1 shows that the amount of precursor used in the Brunauer–Emmett–Teller (BET) process determines the system's surface area. As a result of the synthesized CuO NPs using different extracts of propolis, it was found that they had a surface area of 5.37 to 8.45 m^2^/g for samples 1 and 2, respectively. Propolis extract increased the pore size and decreased the surface area.

### 3.4. SEM and TEM Evaluation

**Figure 4:**
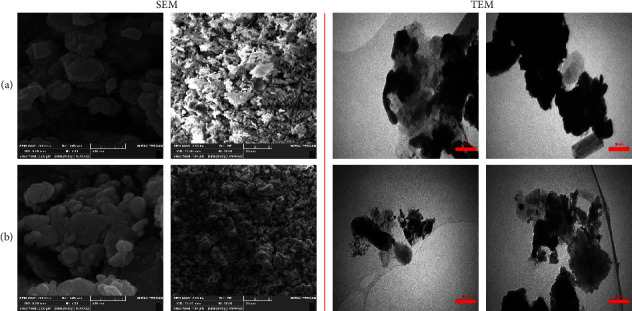
SEM (500 nm and 20 *µ*m) and TEM (50 nm) images of the synthesized CuO NPs of (a) samples 1 (Khalkhal) and (b) sample 2 (Gillan).

Images obtained from SEM micrographs of the synthesized CuO NPs using propolis extract showed that the crystallization structure of CuO NPs synthesized from propolis extract was quite polydisperse and similar to anatase phase CuO crystallites. As a result of the experiments in sample 1 (Khalkhal), NPs with diameters ranging from 75 to 145 nm were observed. There were also NPs ranging in diameter between 120 and 155 nanometers in sample 2. Based on the diameter of the propolis extracts from Gillan and Khalkhal, it was possible to compare the extracts based on their diameters (Figure 4). As can be seen in Figure 4, green CuO NPs were biosynthesized with propolis extracts and taken from Khalkhal and Gillan. In part, the increase in CuO particle size can be attributed to the green biosynthesis among samples 1 and 2. It is important to note that CuO NP particles had narrow sizes and were uniformly dispersed throughout all sides of the particle.

### 3.5. Cell Viability Evaluation

**Figure 5:**
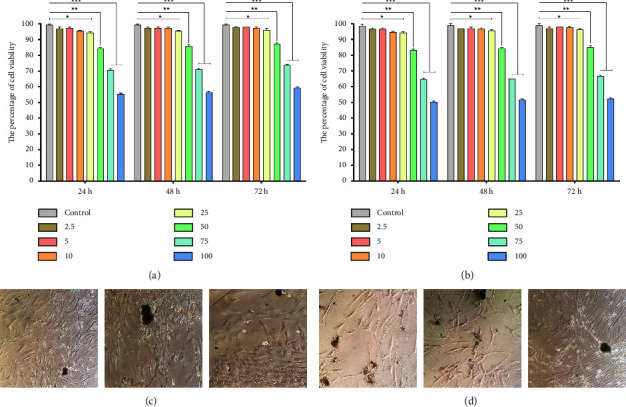
Cytotoxicity test of samples 1 and 2: (a) green synthesized CuO NPs from Khalkhal (sample 1), (b) Gillan (sample 2) (CuO NPs concentration: 2.5, 5, 10, 25, 50, 75, and 100 *μ*g/mL and control: cell culture) (^*∗*^(*P* < 0.05), ^*∗∗*^(*P* < 0.01), and ^*∗∗∗*^(*P*<0.001)), (c) the treated cell culture with CuO NPs sample 1, and (d) sample 2.

Various concentrations of samples 1 and 2 NPs were incubated with HEK cells to determine their effect on the cell-cultured cells (CuO NPs concentration: 2.5, 5, 10, 25, 50, 75, and 100 *μ*g/mL and control: cell culture) for 24, 48, and 72 h. To determine the viability of cells, the cytotoxicity test must be performed. As a result of both the dose and time dependence, the number of viable cells was decreased in samples 1 and 2 NPs. It was determined that the cell viability percentages for the HEK cells and the control groups were calculated for 24, 48, and 72 hours. As a result of MTT data analyses, the cell survival rate of both samples was approximately more than 50% during 24, 48, and 72 hours (Figure 5).

### 3.6. Antimicrobial Analysis

#### 3.6.1. MIC and MBC Results

As a result of broth micro-dilution, MIC of samples 1 and 2 NPs against *L. acidophilus* was determined. The range of MIC values was 0.0024 for *L. acidophilus* (Table 2). As seen in Table 2, levels of MBC were within the range of 0.0024 for *L. acidophilus*.

#### 3.6.2. Biofilm Formation and Degradation Evaluation

**Figure 6:**
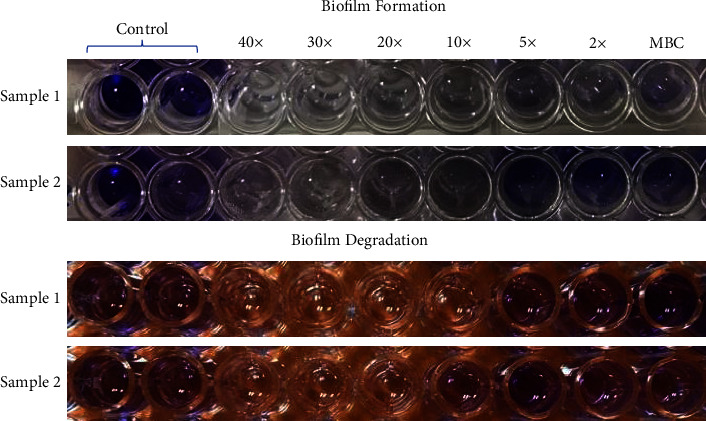
The biofilm formation and degradation tests of samples 1 and 2 NPs. Green synthesized CuO NPs from Khalkhal (sample 1) and Gillan (sample 2).

It was determined that the samples could prevent biofilm formation using microdilution tests as a method of determining their effectiveness.  Table 3 shows the biofilm formation results, and the OD (570 nm) was determined to compare the ODs of treated groups (samples 1 and 2). Sample 2 of the NPs showed the highest levels of biofilm growth compared to sample 1 which showed the highest effect against *L. acidophilus.* Biofilms that had already been formed were also treated with the same method to test the treatment's effects. As a result of the degradation of biofilm, a percentage degradation was calculated (Table 4). There was a significant effect of sample 1 NPs against *L. acidophilus*.  Figure 6 shows the biofilm formation and degradation tests of both NPs against *L. acidophilus.*

## 4. Discussion

Health risks arise from biofilms containing pathogenic microorganisms. Lactobacillus species can colonize tooth surfaces due to bacterial adhesion. Along with mechanical plaque removal, natural antimicrobial mouthwashes enhance it. Chlorhexidine mouthwashes promote tooth decay more than herbal mouthwashes [13]. Global public health has been affected by AMR in recent years. MDR strains of pathogenic bacteria are becoming more resistant to antibiotics due to the emerging trend of AMR. Recently, metallic nanoscale materials have been used more frequently in nanotechnology. The NPs show promising therapeutic effects due to their unique physicochemical properties [36, 37]. In addition, NPs require a thorough understanding of their physicochemical characteristics and appropriate synthesis methods [36, 38]. A new method for synthesizing metal NPs has been developed by utilizing biological cells' highly structured physical and biosynthetic activities. As nanotechnology continues to improve, it is now used in nearly all biomedical applications, from laboratory to large-scale manufacturing. In this study, CuO NPs were synthesized using phase-pure, green propolis extract. Due to phenolic compounds, antimicrobial properties are present in natural materials [39]. In Barbosa et al., silver NPs (AgNP-P) were synthesized from Brazilian propolis and studied for their antimicrobial properties. A factorial design was used to optimize the synthesis conditions for smaller particles. UV-visible spectra revealed that AgNP-P was formed in a spherical structure, with maximum absorbance at 412 nm. This new material shows a good size distribution and a low polydispersity index resulting from dynamic light scattering. Silver NPs were found to contain propolis after centrifugation, and microscopy analysis confirmed it. The principal planes of the metallic silver crystalline structure were identified by X-ray diffraction, while infrared spectroscopy indicated that 22% of the AgNP-P in the sample was reduced to silver. Silver NPs and propolis synergistically demonstrated antimicrobial activity and had significant antimicrobial effects [40].

The CuO NPs were synthesized by Hajizadeh et al. and characterized by FTIR, XRD, and UV-Vis absorption spectra. According to FTIR analysis, propolis extract compounds were found to modify the surface of synthesized NPs. There was a sharp peak at 3422 cm^−1^ in the spectrum of the CuO NPs of the Khalkhal sample. An investigation of UV-Vis spectrophotometry indicates that CuO NPs showed SPR with CuO NPs of the Khalkhal sample at 385 nm. Moreover, CuO NPs of the Gillan sample demonstrated peaks at 243, 292, and 350 nm wavelengths. According to an XRD pattern of the CuO NPs of the Khalkhal sample, the crystallographic planes were 35.74°, 39.04°, and 49.04° and also, 25.54°, 26.69°, 38.79°, and 48.84° were for CuO NPs of Gillan sample [35].

Manikandan et al. report that green-fabricated CuO NPs with a −17.2 mV zeta potential could have more excellent colloidal stability when fabricated from a higher negative charge. NP's catalytic activity is primarily influenced by its surface area, crystal size, and crystallinity. It may be possible for NPs to be highly photocatalytic if the BET value is high, the crystal size is small, and the crystallinity is high. Using propolis increased the rate of photodegradation and specific surface area while decreasing crystal size [41].

According to Moustakas et al., acute CuO NPs exposure caused photosynthetic activity, oxidative stress, and CuO bioaccumulation in seagrasses (*Cymodocea nodosa*). The CuO NPs were characterized with SEM and DLS measurements at 4, 12, 24, 48, and 72 hours (Optimum CuO NPs exposure). In terms of size distribution, CuO NPs synthesized with Pdi 0.35 had an average size of 233 nanometers based on their results [42]. The present study demonstrated that propolis extract could be used to synthesize CuO NPs, but no smaller particles were found. Khalkhal and Gillan propolis extracts were found to interact with CuO NPs in terms of physical interactions.

Green tea (*Camellia sinensis L.*) and lavender (*Lavandula angustifolia*) have been found to synthesize green and pure CuO NPs, respectively. Based on SEM images of lavender-produced samples, it appears that NPs tightly adhered to each other, and lavender was more efficient in generating pure and uniform CuO NPs (50 nm) [43]. A compact distribution of NPs was observed with narrow dispersion. According to the average particle size measurement, NPs were spherical and ranged from 75 to 145 nm for sample 1 (extracted from Khalkhal) and 120 to 155 nm for sample 2 (extracted from Gillan). It has been possible to detect aggregates and NPs of synthetic CuO NPs with spherical shapes on a micrograph.

Veisi et al. studied CuO NPs synthesis with the help of aqueous extracts of *Stachys lavandulifolia* flowers, and they demonstrated that TEM analysis provides morphological information about CuO NPs shapes and dimensions. According to the authors, NPs are synthesized with moderately good monodispersity, with sizes ranging between 15 and 25 nm without agglomeration [44]. As the surface-to-volume ratio decreases, the particle size decreases inversely, facilitating easy penetration of the cell wall and rapid destruction of microorganisms; Khalkhal and Gillan (Khalkhal and Gillan) propolis extract enhanced the antimicrobial activity of green synthesized CuO NPs.

Botteon et al. (2021) described the biosynthesis of AuNPs and evaluated their structural properties. An SPR band was observed at 535 nm in AuNPs. The Brazilian red propolis (BRP) sample used in the reaction affected the sizes and morphologies. All strains tested showed antimicrobial activity against AuNPdichloromethane and AuNPhexane. In both T24 and PC-3 cells, AuNPs showed dose-dependent cytotoxicity. ANPdichloromethane and AuNPextract were the most cytotoxic. Apoptosis-related mechanisms are also responsible for biogenic nanoparticle cytotoxicity [12]. Rao and colleagues employed the coprecipitation method to stabilize and cap Zinc oxide NPs (2018). NPs (PZnO) were synthesized from P betel. PZnO was tested for its antibacterial activity against dental pathogens using a well-diffusion method. Both tested microbes were inhibited by PZnO at concentrations of as low as 3.25 *μ*g/mL, indicating higher antimicrobial activity than DZnO. PZnO and DZnO inhibited cellular growth by 40% using Balb 3T3 mouse fibroblast cell lines [45]. There were no significant cytotoxicity results during the incubation of samples 1 and 2 NPs during the 24, 48, and 72 hours of incubation, which showed that the viability of the samples was approximately more significant than 50%.

NPs synthesized from biosynthetic materials were subjected to MTT assays to assess their impact, and they significantly reduced the survival of cancer cells. NPs synthesized using green methods have been shown to possess antiproliferative properties against cancer cell lines [46, 47]. Silver NPs were green synthesized using leaf extract from *Justicia glauca* by Emmanuel et al. AgNO_3_ solution was mainly reduced to AgNPs by water-soluble organics in leaf extracts. TEM images showed that AgNPs were 10–20 nm in diameter. *S. mutans*, *S. aureus*, *L. acidophilus*, and *C. albicans* were used, and the antibacterial and antifungal activities of AgNPs were demonstrated. AgNPs demonstrated MICs between 25 and 75 g/mL [6]. Based on extracts of *A. javanica* leaf, CuO NPs' antibacterial properties were evaluated against *P. aeruginosa, E. coli, S. aureus*, and *A. baumannii*. There was greater effectiveness of the CuO NPs against *S. aureus*, *P. aeruginosa*, *A. baumannii*, and *E. coli*. According to the sample, *S. aureus* had a maximum inhibition zone of 9 ± 1 mm in the sample. As a result of the extractions with *A. javanica*, *P. aeruginosa* displayed an even more significant inhibition zone. The pathogens that were most active against CuO NPs were *S. aureus, A. baumannii*, *P. aeruginosa*, and *E. coli*, while those with minor activity were *E. coli* and *P. aeruginosa*. It has been demonstrated previously that *S. aureus* was more susceptible to CuO NPs than *E. coli*, based on a comparison between the two samples [48]. There was no difference between samples 1 and 2 in terms of their effect *on L. acidophilus* (0.0024 mg/ml) in the MIC and MBC tests in this study. It was found that the effect of green synthesized CuO NPs from Khalkhal (sample 1) on biofilm formation was more effective than green synthesized CuO NPs from Gillan (sample 2).

## 5. Conclusion

The use of nontoxic, cost-effective, ecofriendly, and easy-to-use materials have proven successful and expanded. All samples were tested for cytotoxicity, and it showed that all samples were free of any notable cytotoxicity. In addition, the results showed that the green synthesized CuO NPs from propolis had antibacterial effects against *L. acidophilus*. It can be concluded that green synthesized CuO NPs derived from propolis may be the best candidate for clinical application due to their high antibacterial properties.

## Figures and Tables

**Table 1:** The specific surface area of different kinds of green synthesized CuO NPs from Khalkhal (sample 1) and Gillan (sample 2).

Samples	Surface area (m^2^/g)
Sample 1	5.3722
Sample 2	8.4537

**Table 2:** MIC and MBC of green synthesized CuO NPs from Khalkhal (sample 1) and Gillan (sample 2).

Bacteria	Sample 1		Sample 2	
	MIC (mg/ml)	MBC (mg/ml)	MIC (mg/ml)	MBC (mg/ml)
*L. acidophilus*	0.0024	0.0024	0.0024	0.0024

**Table 3:** The biofilm formation percentages of species treated (10× MBC) with green synthesized CuO NPs from Khalkhal (sample 1), Gillan (sample 2), and chlorhexidine 0.2% (CHX 0.2%).

Bacteria	OD (570 nm)		CHX 0.2 (%)
	Sample 1	Sample 2	
*L. acidophilus*	6.14%	5.11%	0.76

**Table 4:** The biofilm degradation percentages of species treated (10× MBC) with green synthesized CuO NPs from Khalkhal (sample 1), Gillan (sample 2), and chlorhexidine 0.2% (CHX 0.2%).

Bacteria	OD (570 nm)		CHX 0.2 (%)
	Sample 1	Sample 2	
*L. acidophilus*	82.29%	79.39%	98.34

## Data Availability

The data generated or analyzed during this study are included in this published article.
